# Supplementary figures and images for: Accurate contact-based modelling of repeat proteins predicts the structure of new repeats protein families
Source: PLoS Comput Biol. 2021 Apr 15;17(4):e1008798. doi: 10.1371/journal.pcbi.1008798 (PMC8078820; doi:10.1371/journal.pcbi.1008798)

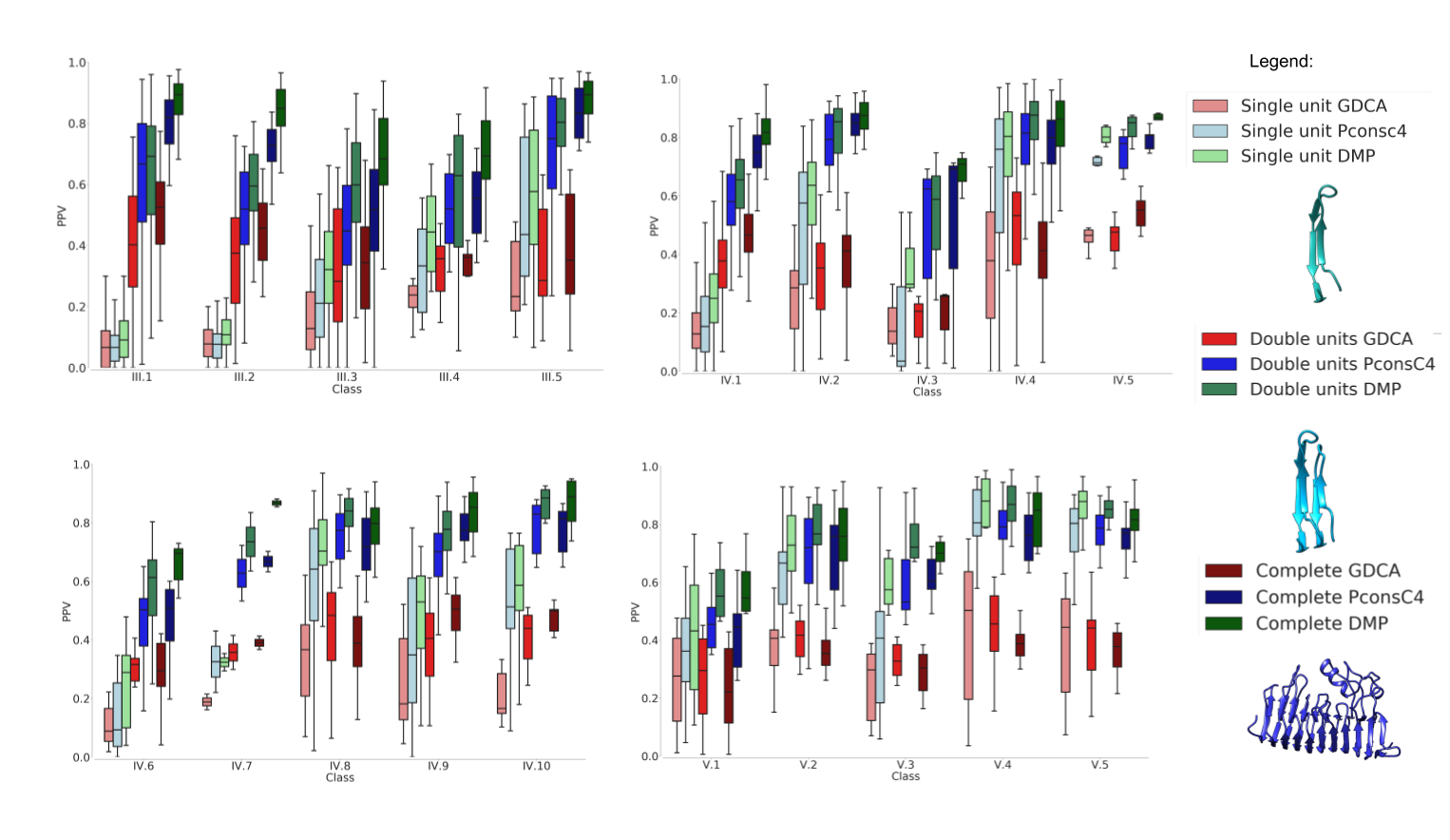

Supplement: S1 Fig — Positive Predictive Value (PPV) for the GaussDCA (red), Pconsc4 (Blue), and DeepMetaPsicov (green). For all three methods results are shown for the three datasets, in light colour the single unit dataset, in intermediate colour the double units dataset, and in the darker colour the complete region dataset. (TIF) [file pcbi.1008798.s001.tif]

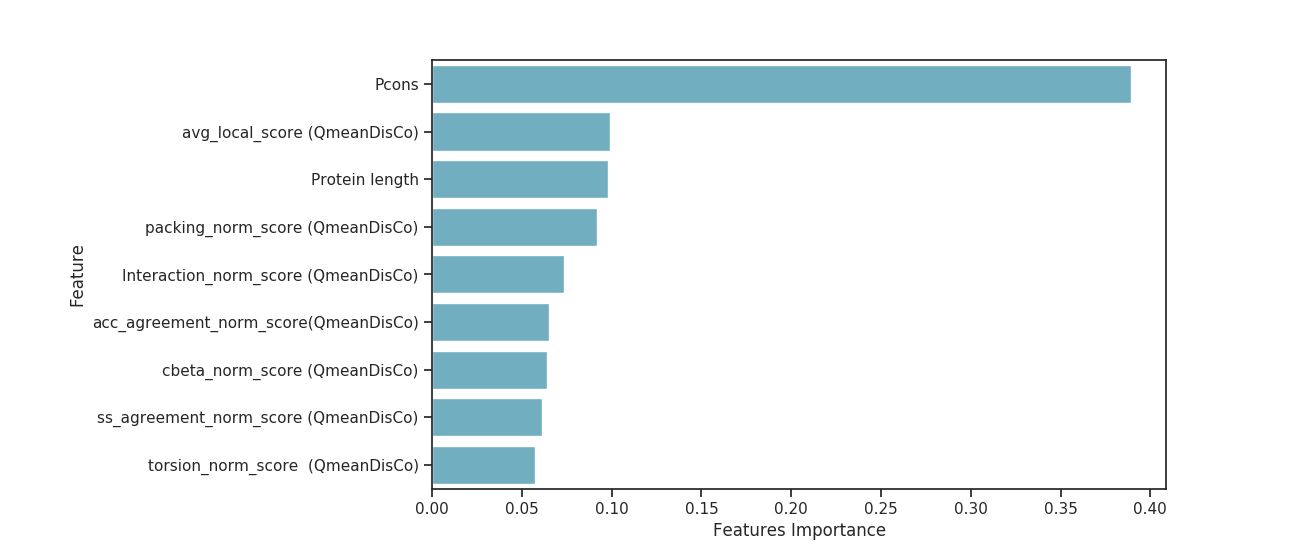

Supplement: S2 Fig — In light green, the Deep Meta Psicov (DMP) average precision for the proteins of the benchmark set not overlapping with the DMP training set, in dark green the DMP average precision for the proteins benchmark set present in the DMP training set, in light blue the PconsC4 average precision for the proteins of the benchmark set not overlapping with the PconsC4 training set, in dark blue the PconsC4 average precision for the proteins benchmark set present as well in the PconsC4 training set. (TIF) [file pcbi.1008798.s002.tif]

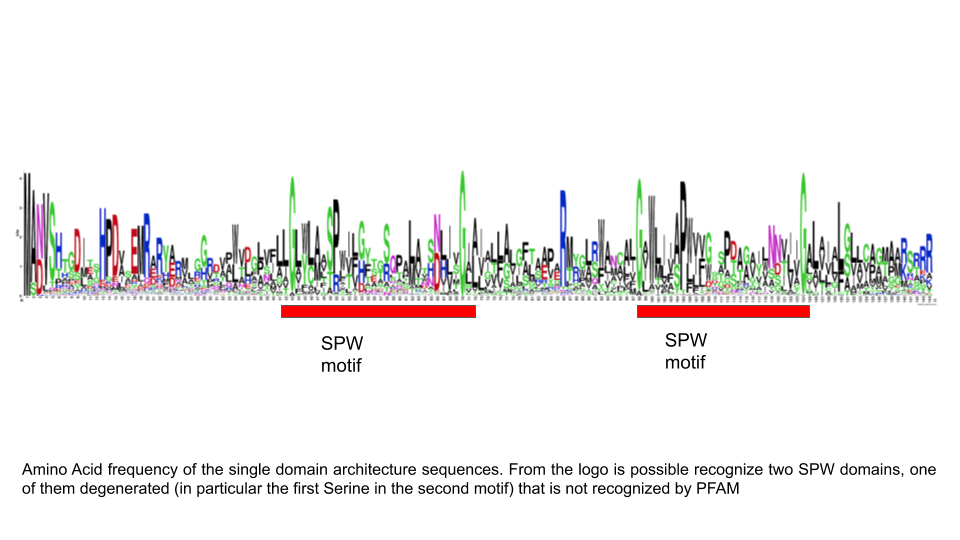

Supplement: S3 Fig — The features used in the random forest model are listed according to their relative importance. (TIF) [file pcbi.1008798.s003.tif]
