# Supplementary material for: Accurate contact-based modelling of repeat proteins predicts the structure of new repeats protein families
Source: PLoS Comput Biol. 2021 Apr 15;17(4):e1008798. doi: 10.1371/journal.pcbi.1008798 (PMC8078820; doi:10.1371/journal.pcbi.1008798)
Supplement: Attachment — Submitted filename: Cover-Letter.docx [file pcbi.1008798.s005.docx]

Dear Editor,

Answering your requirements we expand our paper including predictions made with Deep Meta Psicov however we could not use RaptorX and the method described by reference 17 because the first is available only as web server that does not support , despite is available, is not working at the moment.

The main concern of reviewers 1 and 2 is that, while it is noteworthy that the deep NN learning of contacts allows PconsC4 to model repeat proteins much better than the direct-coupling approach GaussDCA, no comparison to state-of-the-art methods such as RaptorX (Xu lab) or ResTriplet (Zhang lab) or DMPfold (Jones lab) is done. Reviewers 2 and 3 also criticised that no attempt to correct for the specific, systematic noise in contact predictions arising from doublicated repeats, as described in ref (17), has been developed to specifically improve the structure prediction of repeat proteins.

Answer to the reviewers

We thank all the reviewers for their work and suggestions that significantly improved the quality of the article.Here we answer point by point

Reviewer #1:

This manuscript presents the study of deep learning for the structure prediction of proteins with repeats. The authors first used their in-house tool PConsC4 (a deep learning based method for contact prediction) to predict contacts and then apply the predicted contacts to fold the proteins. The authors did a comprehensive analysis of success and failure and claimed that deep learning method works much better than DCA for contact predictions of this specific type of proteins. Overall, the topic addressed in this manuscript is important and interesting and the authors did a nice analysis.

However, there are some concerns:

1) It is unclear if the redundancy between the training data of PConsC4 and the test data has been removed or not. It is important to remove redundancy in order to claim that deep learning works much better than DCA;

Thanks for the suggestion. We examined the redundancy between our repeat datasets and the training sets of PconsC4 and DeepMetaPsicov. There is a small overlap (25 over 2856 PconsC4 and 29 over 3456 DMP). We mention the fact and now in the supplementary we compare the performance of the methods for for the overlapping and non overlapping proteins, showing that there is no difference in the performance.

2) Although PConsC4 is a method that uses a variant of deep convolutional neural network, its performance is much worse than the best in the community, as evidenced in CASP13 and in some recent tests (see<https://academic.oup.com/bioinformatics/advance-article/doi/10.1093/bioinformatics/btz863/5628221> for an example). That is, using PConsC4 to do such an experiment may significantly underestimate the number of repeat proteins that can be folded. Some analysis and conclusion may not be very accurate shall a much better contact prediction program be used. Further, a distance-based folding program may work even better than a contact-based one. Therefore, quantitatively, the analysis presented in this manuscript may not reflect the state of the art.

Thanks for the comment. We repeated the analisis with DeepMetaPsicov significantly improving the results

3) Finally, there is no method advancement in this manuscript.

In the new version of the article we developed a new RandomForestRegression method for assessing the quality of the repeat models, that shows better performance compared with the existing methods.

Reviewer #2:

In the manuscript, the authors present an approach to the prediction of the tertiary structure of repeat proteins. The approach mainly contains two steps, namely, PconsC4 to predict residue-residue contacts of target protein, and CONFOLD to reconstruct tertiary structure based on the predicted contacts. The authors evaluated the approach on a benchmark dataset of 819 repeat proteins. Experimental results suggested that about one third of these proteins could be accurately modelled. The authors further applied this approach to predict structures of proteins in 51 PFAM families without resolved structures.

The experimental results are promising; however, I still have several concerns.

Major concerns:

1. The approach is essentially a pipeline that consists of two existing methods, i.e., PconsC4 and CONFOLD. However, PconsC4 is a general-purpose software for contact prediction, and no specially-designed method was proposed for the repeat proteins. Repeat proteins show outstanding characteristics in both sequence-related features and co-evolutionary features. What is the effect of these characteristics on DCA and subsequent de-noising step using deep learning? How to exploit these characteristics to improve contact prediction and tertiary structure reconstruction?

Unfortunately - decomposing exactly what it is that the deep learning methods use to de-noise the contact maps is difficult. We instead focused on the application of the method on predicting novel structures.

2. In the study, the authors present experimental results of the approach only. It is highly recommended to perform comparison with DMPfold (Greener, et al. Nature Communications 2019).

Thanks for the suggestion we substituted in our pipeline PconsC4 with DeepMetaPsicov (DMPfold) significantly improving the results.

3. The author compared PconsC4 with GaussDCA in terms of accuracy of contact prediction. It is highly expected to compare with state-of-the-art approaches, say RapterX-Contact.

Thank you we repeated the analysis with DeepMetaPsicov (since RaptorX-Contact is avalilable only as web server)

Minor concerns:

1. Grammar errors:

--- Line 50-51: “….divided into 20 subclasses divided by their secondary structure”

--- Line 62-63: “Two subclasses: β-trefoil/β-hairpins, anti-parallel and β-layer/β-hairpins form extended beta strands without the bend typical of the solenoid.” is not a complete sentence.

--- Line 194: “This is observed is regardless of the….”

--- Line 237-238: “the score between the contact-based models and the homology model share a TM-score of 0.58.”

--- Line 252-255: the sentence is too long to follow.

2. Typos:

--- Line 92: “over-perform” => “outperform”?

--- Line 104: “beds on a string” => “beads on a string”?

--- Line 142: “easier do be predicted” => “easier to be predicted”?

--- Line 169: “PSIpred” should be “PSIPRED”

3. Misc:

--- The second paragraph (Line 41-42) of the Introduction section contains only one sentence.

Thankyou for your comments, we corrected the text and made many other grammatical changes.

Reviewer #3:

The authors propose a sequence based approach for modeling the three-dimensional structure of repeat proteins. They show that their previously developed approaches (PconsC4 for contact prediction, Confold for structure modeling, Pcons for quality assessment) allow for producing high-quality models in at least a fraction of repeat-protein families - both via intensive testing in families with known structures, and via prediction of novel structures for families without known structures (successful in about 10% of the tested cases). I think this is a nice and highly publishable work, since the structural modeling of repeat proteins is in fact challenging, and since the paper assesses the full problem from extracting sequences from databases to assessing the quality of the final 3d models.

In general the paper is well written, even if full of little language-related errors - a serious language revision would be important. The current state it is a bit disturbing.

I have few major concerns:

1. They authors compare their approach with DCA, and they see that the non-natural predicted contact patterns (mostly bands at the period of the repeat length, due to the close phylogenetic relation between many neighboring repeats) are successfully classified as non-contacts by the CNN in PconsC4. The authors cite [17] for the fact that DCA has problems with these signals, which appear as spurios coevolutionary signals, but they do not consider that [17] actually proposed a solution for this problem by downweighing pairs of reapeat of high similarity between the repeats (i.e. most likley emerging from relatively recent duplications). Even a very simple filter might remove these bands - while standard contact prediction does not take into account predicted contacts at sequence separation below 5 residues, for repeat proteins also pairs close to (i,i+L) should be excluded, with L being the length of a single domain. I have little doubt that PconsC4 reaches higher accuracy as has been shown by the last author's lab before in more standard protein domains, by I think that the comparision should be rather to [17] instead of GaussDCA.

We try to run the method presented ad [17] however despite available, it work only on very short repetition so is not really comparable with GaissDCA, PconsC4,DeepMetaPsicov. Moreover in supplementary of [17] is possible appreciate that the method improve the DCA prediction only in a limited subset of repeat proteins.

2. Even if below Pcons scores of 0.4, I think the authors should provide structural models also for the other repeat protein families. According to Fig. 7, there are models with TM scores above 0.5 also for Pcons scores around 0.1-0.2, and there are some models with low Pcons scores above 0.4, but rather low TM scores. It could therefore be valuable for researchers interested in these proteins, to have predictions even if possibly of quite low quality - evidently indicating the Pcons score to draw attention to the probably limited model quality.

We develop a new random forest method for quality assessment and we provided ALL the models in 10.6084/m9.figshare.9995618

To these major concerns, a number of minor ones have to be added:

i) Lines 59-68: In the descriptions of the different classes of repeat proteins, it would be good to have numbers for the lengths of the domains etc.

We added the length of the units in the description

ii) Lines 87-88 and Methods: How are the double unit datasets constructed? I guess it is consecutive units, but is the second unit of one sequence counted as the first unit of the next sequence (i.e. if we have units (1,2,3,4,...), do the authors count unit pairs (1,2), (2,3), (3,4) or less redundantly (1,2), (3,4))? In the complete-region dataset, how is the variable number of repeated units taken into account. I would guess that alignments can be shifted.

For the single unit dataset we take only a single unit for protein (in general the central one because is more conserved), the double unit dataset is made with the same single unit protein extended to the following or previous one (chosen by the absence of insertion).

In the complete dataset there is no correction by the unit number. The overall quality of the allignment appear good since the contact prediction on the benchmark set has the expected precision.

iii) Again in the complete-sequence dataset, are there contact between units (i,i+2) or even further away? Or are all contacts strictly inside and between directly consecutive units? Are contacts inside each unit treated independently in the analysis, or is the repeated structure taken into account in calculating PPV? Idem for inter-unit contacts?

In the complete dataset all the contacts among all the units are taken into account between all units. In the inter-unit contact analysis the contacts of each protein were divided in between: “intra” the contacts inside each unit of the protein so in case of units (1,2,3) the protein inside respectively unit 1, + unit 2 + unit 3; “inter“ all the contacts between different units so 1-2, 2-3, 1-3.

iv) Line 100: Is PconsC4 trained on intra-protein contacts in a training set not containing repeat proteins? Or is there some potential non-empty intersection between training and test sets?

Thank you for the comment. We examined the redundancy between our repeat datasets and the training sets of PconsC4 and DeepMetaPsicov. There is a small overlap (25 over 2856 PconsC4 and 29 over 3456 DMP). We mention the fact in the paper and we add in the supplementary comparison between the overlapping and non overlapping proteins, there is no difference.

v) Line 120: First appearance of Neff - please explain. By the way, the explanation in the caption of Fig. 4 is not clear.

Thanks for the comment We clarify the Neff score description and put the reference at the HHsuite manual. We also clarified the caption.

vi) Line 160: The comparison between alpha-helices and beta-strands is interesting, but no empirical data support is given. Is this a general tendency of PconsC4 since CNN are more efficient on beta-strands due to their particular local contact patterns?

Exactly the local pattern of the beta-strands appears to be easier picked by the CNN. However now we show the result for DeepMetaPsicov instead of PconsC4

I have no particular remarks on the discussion of the class-specific results, since I lack familiarity with repeat proteins. So it looks good to me as it is.

vii) Line 300: Sequences are clustered at 40% seq identity, which is quite large. How sensitive is the size of the dataset to clustering at lower identities? How well conserved are repeat protein structures at 40% sequence identity?

At lower identity the dataset does not shrink too much indeed 40% identity the dataset result of 822 proteins (815 after the removal of special case such artificial proteins, retracted PDB ecc...), while there are 706 at 30% and 560 at 20%. The 40% in our dataset correspond to approximately a TM score of 0.9

Overall, I think the paper is interesting and clear, but might benefit in particular from a better comparison with prior work on combining co-evolution based contact prediction and repeat proteins. All other remarks should be addressed very easily.
