# Supplementary material for: Accurate contact-based modelling of repeat proteins predicts the structure of new repeats protein families
Source: PLoS Comput Biol. 2021 Apr 15;17(4):e1008798. doi: 10.1371/journal.pcbi.1008798 (PMC8078820; doi:10.1371/journal.pcbi.1008798)
Supplement: Attachment — Submitted filename: Answer to the editor.docx [file pcbi.1008798.s006.docx]

Dear Editor,

We significantly revisit the manuscript as required by the reviewers. In particular, we thank the reviewers for the suggestion to use trRossetta that dramatically improved the predictions allowing us to solve correctly around 90% of the proteins in both our datasets. In particular the 85% of the unresolved PFAM families. For this reason, we reshape the paper for using trRosetta as the main method and keeping the previous for the benchmark.

We didn’t run RaptorX as require from reviewer 2 because the job policies reported here <http://raptorx.uchicago.edu/ContactMap/> (eg. max 10 protein for each submission, max 500 protein pending, the time required for the prediction) would have made impossible obtain the result for 900 proteins in time.

We believe that thanks to the reviewer suggestion the quality of the paper improved significantly. Here are our point-by-point replies:

**Comments to the Authors:**

**Please note here if the review is uploaded as an attachment.**

Reviewer #1: The paper deals with an interesting and novel aspect of protein feature predictions. The authors took into considerations all the major criticisms raised by reviewers and edited the paper accordingly. The problem of redundancy is now correctly described.

*Thanks*

Reviewer #2: The authors did some modifications of the manuscript by testing the contacts predicted by DeepMetaPSICOV and developing a random forest regression method to predict the quality of a 3D model. However, the authors did not test folding of distance (and orientation information used by trRosetta). It is unclear why the authors did not test the distance (and orientation) information predicted by RaptorX-Contact simply because it is a web server. In fact, it is much more convenient to use a web server than a standalone software package. A hassle-free strategy is to submit the test proteins to the RaptorX-Contact web server and then evaluate the contact/distance prediction and 3D models returned by the server. Overall, this manuscript represents a simple application of existing (but not very cutting-edge) techniques to an interesting problem. As a proof of concept, it may be sufficient, but it may not represent what we can do on the modeling of repeat proteins using existing techniques and tools.

*We have now included trRosetta models, but not RaptorX as the response time for the web server is too slow to allow easy modelling of 800 families. Further, in the tests we have done as well as results from CASP14 trRosetta is superior to Raptor-X.*

Reviewer #3: I find that the paper does not features works of 'exceptional significance', but a specific evaluation of distinct methods to asses a specific technical question. The exceptional significance would be if the paper fully describe and asses novel structures for repeat proteins based on these findings, but I that this is lacking in the manuscript.

*We now provide in total the structure of 41 new families (out of 48 available). We agree that it would be interesting to investigate these in detail - but we do think it is beyond the goals of this paper and do encourage others to use these models to investigate them further.*
